# Re-emergence of *Lilium callosum* Sieb. et Zucc. in Taiwan after a fire allows propagation and renews the possibility of conservation

**DOI:** 10.1186/s40529-017-0202-x

**Published:** 2017-11-13

**Authors:** Ying Chun Chen, Yang Jung Huang, Chiu Mei Wang, Chin An Chiu, Huey Ling Lin, Pei Fang Lee, Ya Ming Cheng, Chen Chang

**Affiliations:** 1 Department of Horticulture, National Chung Hsing University, Taichung 402, Taiwan; 2 Department of Biology, National Museum of Natural Science, Taichung 404, Taiwan; 3 Experimental Forest/Department of Forestry, National Chung Hsing University, Taichung 402, Taiwan; 4 Department of Biotechnology, Fooyin University, Kaohsiung 831, Taiwan; 5 Department of Agronomy, National Chung Hsing University, Taichung 402, Taiwan

**Keywords:** Extinct lily, Propagation, Karyotype analysis, Embryo rescue, Internal transcribed spacer, Near extinction, Conservation

## Abstract

**Background:**

*Lilium callosum* is native to Taiwan, but little is known about it since it has been considered extinct since 1915. After the rediscovery of this rare species after a fire in 2011 in Tunghsiao Township, intensive work has been conducted to count the number in the wild population, to develop a conservation strategy, and to understand its reproductive characteristics and even economic potential.

**Results:**

To conserve the germplasm of this population, three scales from a wild *L*. *callosum* plant were collected to establish a mass propagation system. Flowers from two regenerated plants were crossed by hand-pollination, the ovules were rescued and cultured in vitro, and 10 offspring were obtained. The karyotype was determined to be 2n = 2x = 24 = 2m + 2m(sat) + 2sm + 8st + 10t. The phylogenetic analysis using ITS sequences revealed that the sample of *L*. *callosum* from Taiwan was not grouped with the other accessions of *L*. *callosum* from other regions. The native habitat is classified as grass-dominated vegetation at the early successional stage and a subtropical monsoon-type climate. To clarify the causes of population scarcity in the native environment, reproductive characteristics of regenerated plants were investigated.

**Conclusions:**

Based on the information from this study, it is possible that factors intrinsic to *L. callosum* could combine to limit pollination and seed formation. The *L*. *callosum* pollen only germinated at a temperature that was higher than the native environment, the plants are self-incompatibile, there was a and scarce population, scattered flowering time and dichogamy. Through the culture of these wild harvested parts, the diversity of the germplasm has been broadened and is now available to preserve this rare and valuable species for the future.

**Electronic supplementary material:**

The online version of this article (10.1186/s40529-017-0202-x) contains supplementary material, which is available to authorized users.

## Background

The genus *Lilium* (L.) comprises approximately 100 species inhabiting the northern hemisphere on the continents of Asia, Europe, and North America, including the Asian tropics (latitude 10°–60°) (van Tuyl et al. 2011). These monocotyledonous perennial herbs possess unsurpassed beauty, which means that lilies are of commercial significance for use as cut flowers and garden and potted plants in the worldwide horticultural bulb trade (van Tuyl et al. 2011; Rong et al. 2011). Taiwan has a subtropical island maritime climate and is located in the southernmost reaches of the original habitat of the lily family. Endemic lily species have evolved for the specific climate, including high temperature and humidity in summer and a rare winter temperature lower than 5 °C. *Lilium formosanum* Wallace, *L*. *longiflorum* Thunb. var. *scabrum* Masam, *L*. *speciosum* Thunb. var. *gloriosoides* Baker, and *L. callosum* Sieb. et Zucc. are all species native to Taiwan (Ying 2000). Each species possesses unique qualities that merit ecological and economical interest.

*Lilium callosum* is native to Taiwan, but little is known about it since it has been considered extinct in the wild since 1915. In the early twentieth century, there were only 2 specimens of *L*. *callosum* in Taiwan. Researchers rediscovered the species in 2011 in Tunghsiao Township, renewing the ability to study and conserve this species.

While the rediscovery of this rare species is exciting, intensive work is needed to count the number in the wild population, to develop a conservation strategy, and to understand its reproductive characteristics and even economic potential. As a start, we investigated the native habitat of *L*. *callosum* in Taiwan, increased the population by in vitro scale culture, and evaluated its germplasm characteristics by karyotype analysis and determined the sequence of its internal transcribed spacer (ITS). We improved the cultivation technique, resulting in flowering plants, which were carefully observed, and overcame reproductive obstacles by in vitro ovule rescue. Through these multiply lines of inquiry, this rare *L. callosum* accession has been preserved. Furthermore, we explored possible reasons behind its near extinction in Taiwan, knowledge which can be used to develop conservation strategies for the future.

## Methods

### Habitat survey

To understand the habitat where the *L. callosum* was rediscovered in Taiwan, we explored the accompanying vegetation and ecological climate. We extracted the monthly temperature and precipitation data from the interpolated database (Chiu et al. 2009) to draw the ecological climate diagram. Soil collected from the native habitat was analyzed by the Soil Survey and Testing Center, National Chung Hsing University, Taichung, Taiwan.

### Scale culture, plant regeneration, and acclimation

Three scales were taken from an *L*. *callosum* plant growing in its original habitat (Fig. 2b), allowing the mother plant to remain in its native region. Scales were washed in running water, rinsed twice with 70% ethanol, surface-sterilized with 6% sodium hypochlorite (NaOCl) and two drops of Tween-20 for 20 min, and then rinsed three times with sterile distilled water. Scales were sliced and placed on 10 mL of BM culture medium in 20 × 150-mm test tubes (Pyrex, No. 9820). The BM medium is a modified MS basal medium (Murashige and Skoog 1962) containing 0.5 mg/L niacin, 0.5 mg/L pyridoxine HCl, 0.1 mg/L thiamine HCl, 100 mg/L *myo*-inositol, and 170 mg/L NaH_2_PO_4_, solidified with 0.8% agar (Fei Kung, Taiwan), supplemented with NAA (α-naphtaleneacetic acid, 0.1 mg/L), BA (N6-benzyladenine, 0.1 mg/L), 1 g/L casein hydrolysate, and 3% sucrose and adjusted to pH5.7 with 1 N NaOH before autoclaving at 121 °C for 20 min. Explants were maintained at 25 ± 1 °C under a 12-h photoperiod at a light intensity of 5.6 μmol m^−2^ s^−1^ (daylight fluorescent tubes FL-30D/29, 40 w, China Electric Co, Taipei, Taiwan).

After 4 week of inoculation, explants were subcultured to a fresh medium and kept in the above growth conditions. Bulblets, developed from small explants after 10 week of culturing, were transferred to LD4 medium, consisting of BM medium as described above but without the BA and with 1 g/L activated charcoal. The conditions of the cultures were similar to those described previously, with a light intensity of 55.6 μmol m^−2^ s^−1^. After being subcultured in the LD4 medium for three intervals of eight-weeks, plantlets had bulb circumferences of 1–2 cm with 4–6 leaves. The plantlets were acclimated and planted in plastic pots (7.5-cm diameter) containing peat moss (BVB substrate, the Netherlands), and grown in a net house. Plant and flower size were measured.

### Karyotype analysis

Newly grown root tips (0.5 cm in length) were collected from bulbs of in vitro cultures 7 days after subculturing to the new medium. The root tips were immersed in pretreatment solution (0.2% 1-Bromnaphthalin, 0.25% DMSO) for 7 h at room temperature in darkness. The root tips were removed from this solution and fixed in Carnoy’s solution I (ethanol:acetic acid = 3:1) (Sharma and Sharma 1965) at 5 °C overnight. For chromosome observation, the root tips were macerated in 1 N HCl for 10 min at 60 °C, washed, and stained using Feülgen solution (0.25 g in 50 mL deionized water; HSHIN Instrument, Taiwan) at room temperature in darkness for 1 h. Root tips were then washed twice with deionized water, digested by an enzyme solution (2% cellulose, 5% pectinase, 4 mM citric acid, 6 mM sodium citrate) for 10 min at 37 °C, and then squashed on a glass slide. Chromosome numbers, size, and shape were evaluated for three individual root tips that produced clear slide images. Haploid idiograms were constructed by measuring the average length of each pair of homologous chromosomes and arranging the pairs of chromosomes in descending order (Gao et al. 2012).

### DNA extraction and internal transcribed spacer (ITS) sequence analysis

Total genomic DNA was extracted from leaves of in vitro cultures of *L. callosum* using the Plant Genomic DNA Purification Kit (GENEMARK Technology, Taiwan) according to the manufacturer’s protocol. The entire internal transcribed spacer sequence of the nrDNA region was amplified using the forward primer (5′-CTCCTCCGCTTATTTATATGC-3′) and the reverse primer (5′-TAGGTGAACCTGCGGAAGGATCATT-3′). The polymerase chain reaction (PCR) was executed in a 20 μL solution containing 200 ng genomic DNA template, 1X Taq buffer (Invitrogen Life Technologies), 0.1 mM dNTP, 5 μM for each primer, 3 mM MgCl_2_, and 0.5 units Platnum Taq polymerase (Invitrogen Life Technologies). Amplification was performed in a DNA thermal cycler (GeneAmp PCR system 9700) that was programmed for initial DNA denaturation at 94 °C for 8 min, followed by 32 cycles of denaturation at 94 °C for 1 min, primer annealing at 52 °C for 1 min, and extension at 72 °C for 2 min, and a final extension at 72 °C for 8 min. The PCR products were confirmed on 2% agarose gel, and then cloned into the pGEM-T easy vector (Promega) and sequenced by Tri-I Biotech, Inc. (Taipei, Taiwan). The sequences were deposited into the NCBI sequence database (National Center for Biotechnology Information, GenBank).

The nuclear ribosomal spacer regions, ITS1 and ITS2, and the 5.8S ribosomal gene (ITS) of *Lilium* spp. were obtained from the NCBI sequence database for phylogenetic analysis, and the sequence of *Fritillaria pallidiflora* was used as an outgroup taxon. DNA sequences were aligned using CLUSTALW (Thompson et al. 1997), and phylogenetic relationships were analyzed by PAUP_v. 4.0.b10 (Swofford 2002). Parsimony analyses were conducted using a heuristic search strategy. For assessing the relative robustness for branches, the bootstrap method (Felsenstein 1985) was used with 1000 replicates, and groups were retained when bootstrap percentages ≥ 70. The trees obtained in these analyses were drawn with the TreeGraph 2 software (Stover and Muller 2010).

### In vitro pollen viability test, embryo rescue and offspring cultivation

Pollen from net house grown plants were harvested and analyzed by SEM. Pollens were fixed, dehydrated, dried in a critical point dryer (HCP-2, Hitachi, Japan) and then coated with gold by an ion coater (E1010, Hitachi, Japan). After preparation, samples were examined by a Hitachi S-3000 scanning electron microscope (Chang et al. 2005). At anthesis, in vitro pollen germination was investigated by placing pollen grains on BK medium (Brewbaker and Kwack 1963) with 5 or 10% sucrose at 15–30 °C. For pollination compatibility examination, anthers were collected before the date of flowering and stigma were covered by aluminum foil and then hand pollinated after 3 days of blooming. The swollen ovary were collected 14 days after pollination and sterilized with 75% ethanol for 1 min, 1% NaOCL with 1 drop Tween-20 for another 10 min, and washed by distilled water three times. The ovaries were cut crosswise into 3–4 mm discs and cultured in the dark at 25 °C on BM basal medium supplemented with 9% sucrose and 1.0 mg/L NAA, with pH adjusted to 5.8. When the ovules became tumescent, they were picked and subculture on BM medium with 5% sucrose, 1 g/L casein hydrolysate, 1.0 mg/L NAA, at pH 5.8.

### Component analysis of bulb scales

After anthesis, the bulbs were harvested and scales were removed to analyze water content, water extractable matter, and alcohol soluble matter, following the Chinese Pharmacopoeia commission’s protocol (2010) with minor modification. The bulb scale sample (3 g) was placed in a glass petri dish and dried in an oven at 105 °C for 5 h. The lid was replaced and the dish was cooled at room temperature for 30 min, weighed and recorded. The sample was dried again at 105 °C for 1 h, cooled and weighed. The measurement was repeated until two consecutive weightings did not differ by more than 5 mg. Percentage of water content was calculated from the weight loss. The water-soluble and alcohol-soluble extract contents were surveyed using a cold-soak method. A bulb scale (1.2 g) was placed in a 50-mL Erlenmeyer flask to which 30 mL of water (or 70% ethanol for alcohol soluble matter) was added. The flasks were covered with aluminum foil and shaken at 125 rpm for 6 h, then allowed to stand for 18 h. The sample was percolated through 90 mm (Whatman #1) filter paper. The filtrate (10 mL) was dried at 105 °C for 3 h in an aluminum pie pan then moved to a dryer to cool for 30 min. The sample was promptly weighed to calculate the water-soluble matter (or alcohol-soluble matter) of the bulbs. The components of bulb scales from *Lilium callosum*, three other native Taiwan *Lilium* species, and commercial, edible and medicinal lilies were compared.

## Results

### Habitat survey

After an accidental fire on a slope near the seaside in Tunghsiao Township, Miaoli County, Taiwan in 2011, researchers found five native *L*. *callosum* plants. The habitat was grassland on a northwestern slope at an altitude of only 130 m. The flowers, seen in September, were orange in color, with reflexed perianths (Fig. 2a). In the natural habitat, the plant most highly associated with *L. callosum* was *Arundinella hirta* (Thunb.) Tanaka, followed by *Paspalum orbiculare* G. Forst., *Cymbopogon tortilis* (Presl) A. Camus, *Setaria glauca* (L.) P. Beauv., and *Rhus chinensis* Mill. var. *roxburghiana* (DC.) Rehd (see Additional file 1: Appendix S1).

**Fig. 1:**
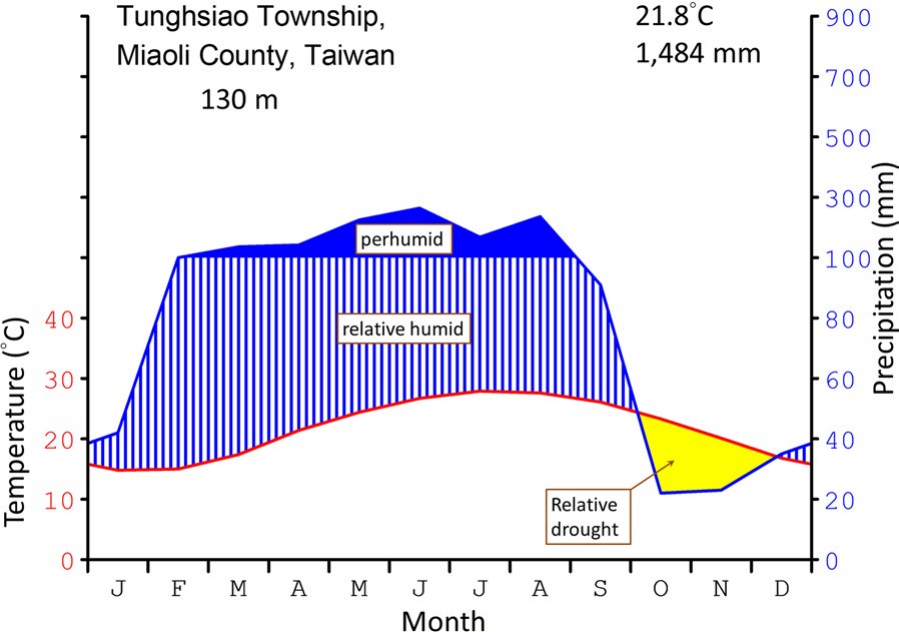
Ecological climate diagram of the *Lilium callosum* habitat

The overall physiognomy of the habitat was grass-dominated vegetation at the early successional stage. The monthly mean temperature of the habitat ranged from 14.8 to 27.9 °C, and the monthly precipitation ranged from 22 to 264 mm. Rainfall and temperature patterns revealed a subtropical monsoon-type climate that is warm and moist but has a relatively dry period during the winter (Fig. 1). The soil contained 4.39% organic matter, 0.176% total nitrogen, 2.87 mg/kg Bray-1 phosphorus, 280 mg/kg exchangeable calcium, 146 mg/kg exchangeable potassium, 104 mg/kg exchangeable magnesium, 16.0 mg/kg exchangeable sodium and had a pH of 5.2. The soil was strongly acidic and rich in organic matter. The area is close to the sea, and the plants in habitat showed fluctuations in phase and species.

In 2011, there were no more than five flowering plants observed, and the number visible by survey has decreased each year thereafter. The fire exposed the lily to sunlight, allowing the plants to bloom. During the first 4 years after the fire, the other plants in the habitat also grew and covered the area, hindering both the bloom of *L*. *callosum* and the researchers’ search for *L*. *callosum* in flower.

### Scale culture, plant regeneration, and acclimatization

**Fig. 2:**
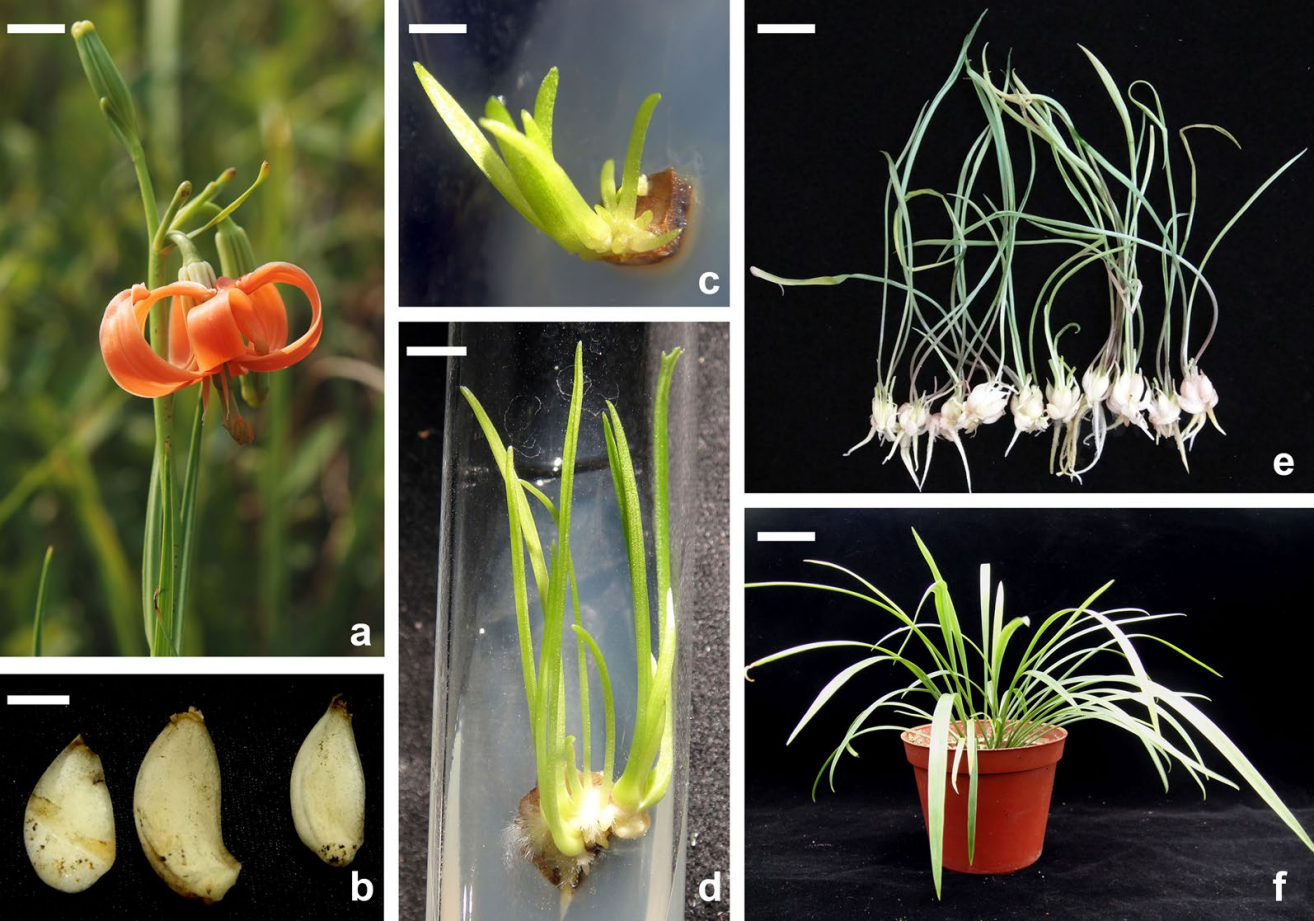
Flower morphology, scale culture, and plant regeneration of *Lilium callosum* found in Taiwan. **a** Flower of *Lilium callosum* in native habitat in Tunghsiao Township, Miaoli County, Taiwan. (Bar = 1.6 cm). **b** Scales collected from wild plants served as explants for in vitro culture. (Bar = 0.7 cm). **c** Small bulblets regenerated from scales after 8 weeks of culture. (Bar = 0.3 cm). **d** Leaf growth and rooting of bulblets. (Bar = 0.5 cm). **e** Well-grown bulbs were taken out of the test tube, analyzed, and transplanted into a pot. (Bar = 1.5 cm). **f** Transplanted plants were set in a net house and grew numerous leaves. (Bar = 3.5 cm)

Three bulb scales taken from wild plants were sliced into 25 pieces. The pieces formed callus from the inner cells or from the incision after 30 days of culturing in vitro. The percentage of callus induction was 20%. All calli turned green. Granules developed from the green calli, and the granules then developed into complete bulbs with shoots and roots (Fig. 2c, d). The average fresh weight of the plants was 0.96 g, average bulb circumference was 1.6 cm, and average leaf count was 4.8 leaves per bulb. We removed 17 bulbs from the test tubes (Fig. 2e) and then transplanted these bulbs to the net house (Fig. 2f).

**Fig. 3:**
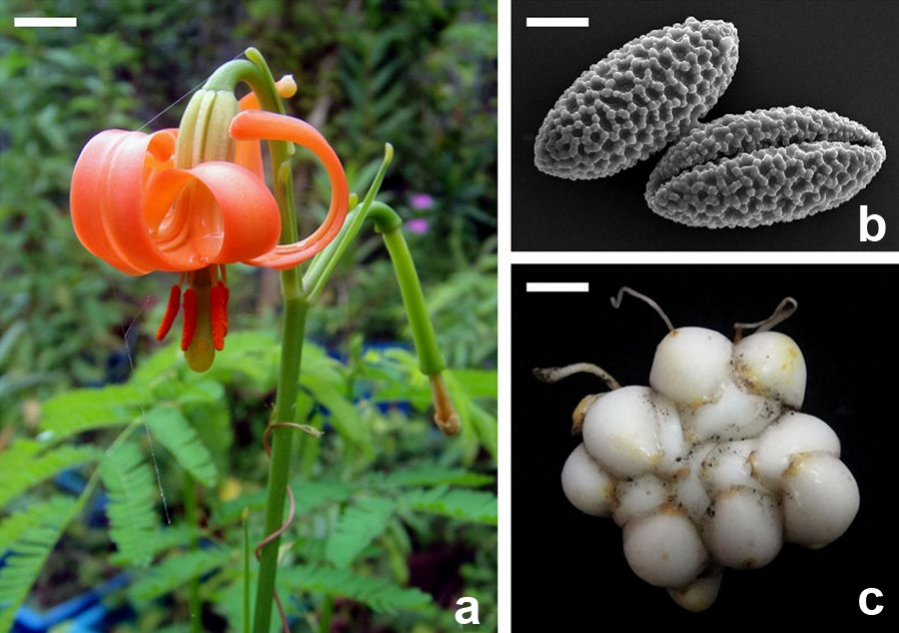
Flower and bulb morphology of regenerated *Lilium callosum* derived from scale culture in vitro. **a** Orange flower color, reflexed perianths, stigma, and anthers containing pollen showed structures similar to those observed in situ. (Bar = 0.7 cm). **b** Scanning electron microscopy showed spindle-type pollen, with a groove and patterns. (Bar = 17.85 µm). **c** A mature bulb with flowering potential has multiple layers of light-yellow or white scales. (Bar = 2.6 cm)

After 15 months of culture, the maximal weight was 18.8 g per bulb and the maximal bulb circumference was 13 cm. We examined a total of six flowering plants and calculated an average plant height of 72.5 ± 10.4 cm, an average leaf number of 33.5 ± 5.9, and an average number of flower buds of 1.8 ± 0.7. The flower morphology was similar to that of the wild plants: orange, reflexed petals (Fig. 3a), with average lengths for the outer and inner perianths of 5.8 ± 0.6 cm, for the style of 1.2 ± 0.08 cm, for the ovary of 3.1 ± 0.34 cm, for the thrum of 2.4 ± 0.32 cm and for the anther of 0.8 ± 0.19 cm. Mature pollen length was 74.6 ± 2.7 µm and the width was 32.3 ± 1.4 µm (Fig. 3b). Mature bulbs with flowering potential had multiple layers of light-yellow or white scales (Fig. 3c).

### Karyotype analysis

**Fig. 4:**
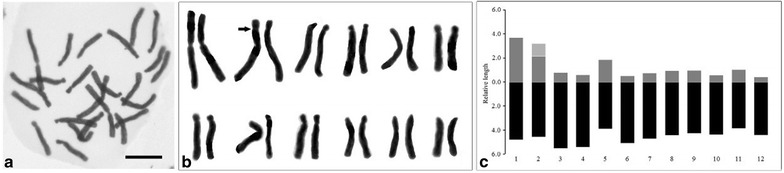
Karyotype analysis of *Lilium callosum*. **a** Somatic mitotic metaphase chromosomes (2n = 24). (Bar = 5 μm). **b** Karyogram. Arrow indicates the secondary constriction. **c** Idiogram. The relative length of each chromosome is on the left

Somatic mitotic metaphase cells of *L*. *callosum* with dispersed chromosomes were obtained for karyotype analysis. The diploid chromosome number was 2n = 24 (Fig. 4a). Figure 4b, c display the karyogram and idiogram, respectively. The karyotype formula was 2n = 2x = 24 = 2m + 2m(sat) + 2sm + 8st + 10t. Table 1 displays other detailed features. The combined length of a haploid set of *L*. *callosum* chromosomes was 70.57 μm, and chromosome lengths ranged from 4.81 to 8.49 μm. We discovered a secondary constriction in the short arm of the second pair of chromosomes.

**Table 1:** Characteristics of somatic chromosomes at metaphase in Taiwanese *Lilium callosum*

Chromosome	Chromosome length (μm)^a^			Relative length (%)^b^	Arm ratio (L/S)	Chromosome type^c^
	Short arm (S)	Long arm (L)	Full length			
1	3.68 ± 0.16	4.82 ± 0.29	8.49 ± 0.43	12.03	1.31	m
2	3.20 ± 0.19	4.56 ± 0.09	7.76 ± 0.28	10.99	1.43	m (sat)
3	0.77 ± 0.06	5.51 ± 0.07	6.28 ± 0.10	8.90	7.16	t
4	0.60 ± 0.12	5.42 ± 0.08	6.01 ± 0.17	8.52	9.03	t
5	1.86 ± 0.16	3.90 ± 0.26	5.75 ± 0.35	8.15	2.10	sm
6	0.52 ± 0.10	5.09 ± 0.29	5.60 ± 0.28	7.93	9.79	t
7	0.76 ± 0.07	4.71 ± 0.18	5.47 ± 0.11	7.75	6.20	st
8	0.93 ± 0.14	4.45 ± 0.07	5.38 ± 0.08	7.62	4.78	st
9	0.95 ± 0.13	4.25 ± 0.22	5.20 ± 0.08	7.37	4.47	st
10	0.56 ± 0.09	4.39 ± 0.26	4.96 ± 0.18	7.03	7.84	t
11	1.01 ± 0.21	3.85 ± 0.25	4.86 ± 0.33	6.89	3.81	st
12	0.42 ± 0.06	4.40 ± 0.24	4.81 ± 0.29	6.81	10.48	t

^a^The data is an average from three individuals

^b^Relative length (%) = (chromosome length/total chromosomes length) × 100

^c^The position of the centromeric constriction was recorded as median (m 1.0–1.7), submedian (sm 1.71–3.0), subterminal (st 3.01–7.0), and terminal (t > 7.01). m (sat) median with satellite

### Phylogenetic analysis using ITS sequence

**Fig. 5:**
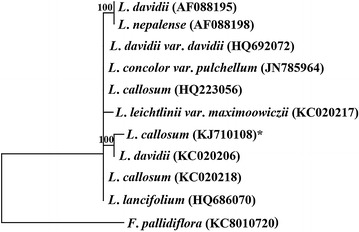
The phylogenetic tree was generated from parsimony analyses using the nuclear internal transcribed spacer (ITS). The tree is rooted with *Fritillaria pallidiflora*, and the values above branches indicate node support (bootstrap percentages above ≥ 70). *Indicated the sequence of *L*. *callosum* in this study

The ITS region of *L*. *callosum* was sequenced and was deposited into the GenBank nucleotide sequence database with the accession number KJ710108. The polycistronic rRNA precursor transcript was 590 bp, with ITS1 ranging from 1 to 229 bp, 5.8S rRNA gene ranging from 230 to 393 bp, and ITS2 ranging from 394 to 590 bp. Sequence alignment for KJ710108 via nucleotide BLAST on NCBI showed 99% identity score with 12 ITS sequences of *L. callosum* from Korea and one ITS sequence of *L. callosum* from China. The phylogenetic tree (Fig. 5) demonstrated that the ten sequences most similar to the ITS sequence (KJ710108.1) of *L*. *callosum* on NCBI website were all belonged to the *Sinomartagon* section, *Lilium*, Liliaceae. Those included *L*. *leichtlinii* var. *maximowiczii*, *L*. *lancifolium*, *L*. *concolor* var. *pulchellum*, *L*. *davidii*, *L*. *nepalense* and two other sample of *L*. *callosum*. The two *L*. *callosum* samples were respectively collected from the Funiu Mountain, Henan Province, China (KC020218.1) and Hoesu-dong, SeogwipoSi, Jeju Province, South Korea (HQ223056.1). Based on the phylogenetic relationship analysis, the ITS sequence of native *L*. *callosum* in Taiwan is clearly distinguishable from *L*. *davidii* (KC020206), grouped equally with the species in the section *Sinomartagon*, but it is not grouped with other two accessions of *L*. *callosum* from different countries. To compare the environmental difference of *L. callosum* habitats, the sample KJ710108.1 (Taiwan) is located at subtropical latitude of ca. 24°N, and KC020218.1 (China) and HQ223056.1 (South Korea) are located at temperate latitude of ca. 33°N.

### In vitro pollen viability test, embryo rescue, and offspring cultivation

Different culture media and temperatures were tested for pollen germination. No pollen germinated in vitro at 15 °C, and only a few pollen germinated on the BK medium with 10% sucrose at 20 °C (data not shown). The highest percentage of in vitro pollen germination was 66.2% when the pollen was cultured on the BK medium with 5% sucrose at 30 °C, followed by a rate of 51.4% when cultured on the 10% sucrose medium at 30 °C (Table 2). Increased sucrose concentration decreased the pollen germination at both 30 and 25 °C. Temperature and sucrose concentration showed coordinated effects on pollen germination (Table 2).

**Table 2:** In vitro germination of fresh *L. callosum* pollen on the BK medium with different sucrose concentrations and under different temperatures

Temperature	Sucrose concentration (%)	Pollen germination percentage (%)^z^		
		4 h	8 h	12 h
25 °C	5	0.19 b	0.00 c	5.09 c
	10	0.00 b	0.00 c	0.00 c
	15	0.00 b	0.21 c	0.00 c
30 °C	5	9.38 a	54.68 a	66.18 a
	10	6.49 a	24.93 b	51.40 b
	15	0.74 b	2.93 c	6.76 c
Correlation analysis^y^	Temp	***	***	***
	Suc	***	***	***
	Temp × Suc	**	***	***

^z^Average of data collected from three individuals. Different letters within a column indicate significant differences at *p* = 0.05 by LSD test

^y^ **, *** Mean that the different letters within a column are significantly different at p > 0.01 and > 0.001, respectively

**Fig. 6:**
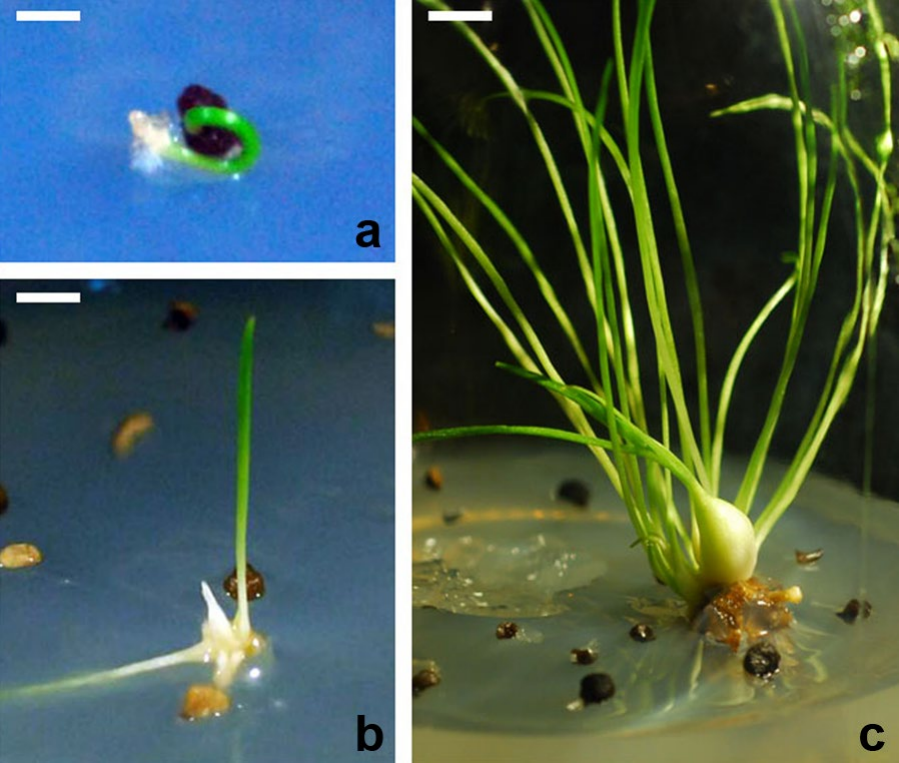
Germination and plantlet formation from in vitro-rescued ovule of *Lilium callosum.* **a** In vitro-rescued ovule germination. (Bar = 0.24 cm). **b** Cotyledon elongation and further growth of in vitro-rescued ovule. (Bar = 0.55 cm). **c** Plantlet formation from in vitro-rescued ovule. (Bar = 0.87 cm)

After selfing by hand pollination, the ovary swelled, but there was no significant growth within 1 month. Anatomical observation revealed that the ovule was swollen but that seeds failed to develop. Two native plants were cross-pollinated by hand. Ovaries were collected 14–16 days after cross-pollination and were sliced into discs and placed on the BM medium supplemented with 9% sucrose and 1.0 mg/L NAA. The ovules became swollen after 14 days. The cultured ovules turned brown 70 days after pollination and were subcultured to new culture medium to obtain germinated plants (Fig. 6). Using this procedure, ten seedlings were obtained for further research and conservation purposes.

### Component analysis of bulb scales

Properties of fresh scales from *L*. *formosana*, *L*. *speciosum* Thunb. var. *gloriosoides* Baker, and *L*. *longiflorum* were compared with those from *L*. *callosum*. The soil-grown *L*. *callosum* sample had the lowest water content, at 66.5%. The water extractable matter in *L*. *callosum* was 46.8%, the highest in all the samples, followed by the purchased edible scales. *L*. *callosum* had the lowest alcohol soluble matter content (Table 3).

**Table 3:** Component of bulb scales among five *Lilium* species

Species	Scale content (%)		
	Water content	Water extractable matter	Alcohol soluble matter
*Lilium formosana*	77.9^a^	37.4	21.2
*L. speciosum* var. *gloriosoides*	79.6^a^	26.5	31.4
*L. callosum*	66.5	46.8	8.1
*L. longiflorum*	79.3	10.5	39.6
Edible lily scales 1	–^b^	43.3	26.4
Edible lily scales 2	–^b^	44.9	30.2
Medicinal lily scales	–^b^	17.1	9.2

^a^Average of data were collected from two bulbs

^b^The dried scales were purchased from stores

## Discussion

A unique opportunity presented itself in 2011, when five plants of *L*. *callosum* were found in an area recently razed by fire. This plant had not been seen in the wild in Taiwan since the first record in 1915. This reemergence illustrated the importance that specific characteristics of bulb dormancy and habitat have on the life of this species. The subtropical monsoon-type climate of this seaside region is at the far reaches of this species’ habitat range, indicating that this Taiwanese ecotype of *L*. *callosum* may be unique. The karyotype and ITS sequence did show that the newly found specimens were unique compared to those native in other regions. The discovery of these plants has allowed investigation into why this species has become nearly extinct in Taiwan and what factors in the habitat may be obstacles to its pollination and reproduction.

To conserve this rare species, we micropropagated more than 1000 plants by scale culture, thin cell layer culture, and leaf culture in vitro, yielding regenerated plants that flowered after cultivation in soil (data not shown). The results indicated that the *L*. *callosum* native to Taiwan has strong multiplication and regeneration abilities, as do other *Lilium* spp. Hence, bulb productivity is not the major cause for the extinction.

*Lilium callosum* is a widespread species in Asia, distributed from Taiwan to northern Russia. Its flowering times are different across the distinct areas. The plant height of *L. callosum* is 40–60 cm in China (Sun 2002) and 70–74 cm in Taiwan (data not shown). The number of leaves present at flowering time in China was less than in Taiwan. Native plants in China (Luanchuan County, Henan, with an average annual temperature of 12.1 °C at an elevation of 500 m) took 65 days from sprouting to visible flower bud, and then flowered after another 16 days (Zhi et al. 2009). In Harbin, China, where the average annual temperature is 0.7–4.2 °C, native plants took 68 days from sprouting to flower bud (Zhao et al. 2010). Under the higher average annual temperature of 21.8 °C and the subtropical monsoon climate in Taiwan, the tissue culture propagated plants took 79 days from sprouting to visible flower bud and 24 days to flower opening (Huang 2016). The *L. callosum* native to Japan initiates a flower bud just after sprouting, and the plants flower after 106 days (Ohkawa 1989). These results indicated that shoot elongation and flower differentiation in *L*. *callosum* is controlled not only by low temperature or stratification, but also by vegetative maturity and high temperature climate in the summer. These influences resulted in the late-flowering observed for this species in Taiwan.

One limit to reproductive success of *L. callosum* in Taiwan was found to be the inability to produce a sufficient number of seeds in the native habitat, which could otherwise increase both the population and the genetic diversity. We found the optimal temperature for in vitro pollen germination was 30 °C, which is higher than the air temperature in September in the native environment. In addition to the limited pollen germination, self-incompatibility, scarce population, scattered flowering time, and dichogamy could be factors that limit the success of pollination and seed formation in *L. callosum* in Taiwan. In this study, we use the strategy of embryo rescue to conserve germplasm. This approach may be important to the conservation of *L. callosum* in Taiwan.

Chromosome numbers of *L. callosum* were 2n = 24, but the karyotype formula and B chromosome number is different among the populations in various areas. Stewart (1947) mentioned that five *L*. *callosum* plants were alike, possessing six chromosome pairs with nucleolar secondary constriction: the first, third, sixth, seventh, ninth, and distal constrictions in the short arm of the second chromosome. For *L*. *callosum* collected in Northeast China, the chromosome numbers were diverse (2n = 22, 24, 24 + 2B, and 26), but 89% of them possessed a chromosome number of 2n = 24, and the karyotype formula was 2n = 2x = 24 = 4m + 2sm + 2st + 14t + 2t(SAT) with a satellite in the ninth chromosome (Yang et al. 1996, 1998). *L*. *callosum* collected from Anhui province, China had a karyotype formula of 2n = 24 = 2m + 2sm + 4st (2sc) +16t, with a secondary constriction in the twelfth chromosome (Shao et al. 1994). The karyotype formula of *L*. *callosum* in this study was 2n = 2x = 24 = 2m + 2m(sat) + 2sm + 8st + 10t, with a secondary constriction in the second chromosome (Fig. 4). The secondary constriction is a useful chromosome feature for identifying a chromosome from a set. On a metaphase chromosome, rRNA genes, which are arranged in a tandem repeated manner, are localized in the nucleolus organizer regions (NORs) and are seen as secondary constrictions (Stępiński 2013). The number and location(s) of secondary constrictions are considered species-specific. The diverse karyomorphology among the *L*. *callosum* populations (Fig. 4), suggested that the Taiwanese *L*. *callosum* can be considered a unique ecotype. These different *L*. *callosum* karyotype formulas could be generated by different geographical paths of the isolated populations. To our limited knowledge, this is the first report of karyotype analysis of *L*. *callosum* native to Taiwan, and the results provide basic information about this scarce species.

The application of molecular techniques, such as DNA sequencing, for phylogenetic analysis has successfully distinguished morphological similarity from homoplasy (Nishikawa et al. 1999). The phylogeny of numerous angiosperms has been investigated using the internal transcribed spacer (ITS) region (Baldwin et al. 1995). Information of ITS sequences has been published for different *L*. *callosum* populations collected from Japan (Dubouzet and Shinoda 1999; Nishikawa et al. 1999, 2001), South Korea (Sultana et al. 2011; Lee et al. 2011), and China (Du et al. 2014). Sequence-related amplified polymorphism (SRAP) markers (Li et al. 2011) and *rbcL* and *matK* gene sequences were also used to analyze phylogenetic relationships between *Lilium* species, including *L*. *callosum*, and related genera (Hayashi and Kawano 2010). All previous studies classified *L*. *callosum* in the section *Sinomartagon* based on the morphological characteristics (Comber 1949) and ITS sequences. In comparison with different origins of *L*. *callosum*, plants from Korea, Japan and China were placed into different groups, and this may be attributed to the considerable divergence in lengths and sequences of ITS1 and ITS2 (Sultana et al. 2011). Pelkonen and Pirttilä (2012) also constructed a phylogenetic tree of the main species and sections of the *Lilium*. *L*. *bulbiferum* and *L*. *dauricum* were grouped with the species in the section *Sinomartagon*, but they were not grouped together. In our study, it is the first study to present an analysis of ITS sequences (KJ710108.1) from the rare population of *L. callosum* native to Taiwan and the result shows an independent lineage with the other accessions of *L. callosum* native to China and South Korea. According to the information of *L. callosum* habitats, the population of *L*. *callosum* from China is found in the thicket slop at an altitude of 565 m and located at temperate latitude of ca. 33°N which has similar environment of the population of *L*. *callosum* in South Korea (Du et al. 2014; Lee et al. 2011). Whereas, this species in Taiwan is located on a grassland on northwestern slope at an altitude of 130 m and at subtropical latitude of ca. 24°N. It is possible that the isolated population and different local climates may lead to the divergence of ITS sequences in various areas.

Lily is a globally important commercial floral crop. The introduction of novel species and ecotypes into breeding programs could expand its uses and value. The pure orange color of the *L*. *callosum* flower is unique and could be useful for ornamental purposes. Huang (2010) mentioned that scales of *L*. *callosum* native to Henan province were edible and used as medicine. We showed that the water extractable matter of *L*. *callosum* in Taiwan was higher than that in purchased medicinal lily scales and was close to that of edible lily scales. Together these results indicated that *L*. *callosum* has potential for both ornamental and edible purposes, although these uses require further investigation.

## Conclusion

The rediscovery of *L*. *callosum* in Taiwan provides valuable materials for conservation, research and horticultural use efforts. Findings in this study not only reveal the reasons behind the mysterious disappearance of this species, but also broaden the diversity of the germplasm available to preserve this rare and valuable species.

## Additional file

**Additional file 1: Appendix S1.** Plants associated with *Lilium callosum* in the Taiwanese native habitat.

## Abbreviations

NAA: α-naphtaleneacetic acid
BA: N^6^-benzyladenine
ITS: internal transcribed spacer

## References

[CR1] Baldwin BG, Sanderson MJ, Porter JM, Wojciechowshi MF, Campbell CS, Donoghue MJ (1995). The ITS region of nuclear ribosomal DNA: a valuable source of evidence on angiosperm phylogeny. Ann Missouri Bot Gard.

[CR2] Brewbaker JL, Kwack BH (1963). The essential role of calcium ion in pollen germination and pollen tube growth. Am J Bot.

[CR3] Chang C, Chen YC, Yen HF (2005). Protocorm or rhizome? The morphology of seed germination in *Cymbidium dayanum* Reichb. Bot Bull Acad Sin.

[CR4] Chinese Pharmacopoeia commission (2010). Pharmacopoeia of the People’s Republic of China 2010.

[CR5] Chiu CA, Lin PH, Lu KC (2009). GIS-based tests for quality control of meteorological data and spatial interpolation of climate data: a case study in mountainous Taiwan. Mountain Res Dev.

[CR6] Comber HF (1949). A new classification of the genus *Lilium*. Lily Year Book RHS.

[CR7] Du YP, He HB, Wang ZX, Li S, Wei XC, Yuan XN, Cui Q, Jia GX (2014). Molecular phylogeny and genetic variation in the genus *Lilium* native to China based on the internal transcribed spacer sequences of nuclear ribosomal DNA. J Plant Res.

[CR8] Dubouzet JG, Shinoda K (1999). Phylogenetic analysis of the internal transcribed spacer region of Japanese *Lilium* species. Theor Appl Genet.

[CR9] Felsenstein J (1985). Confidence limits on phylogenies: an approach using the bootstrap. Evolution.

[CR10] Gao YD, Zhou SD, He XJ, Wan J (2012). Chromosome diversity and evolution in tribe Lilieae (Liliaceae) with emphasis on Chinese species. J Plant Res.

[CR11] Hayashi K, Kawano S (2010). Molecular systematics of *Lilium* and allied genera (Liliaceae): phylogenetic relationships among *Lilium* and related genera based on rbcL and *matK* gene sequence data. Pl Spec Biol.

[CR12] Huang XH (2010). Investigation and development of wild germplasm resources of *Lilium* in Dabie Mountains in Henan province. North Hortic.

[CR13] Huang YJ (2016) Tissue culture of Taiwan native *Lilium callosum* Sieb. et Zucc. and immature embryo rescue. Master thesis, National Chung Hsing University

[CR14] Lee CS, Kim SC, Yeau SH, Lee NS (2011). Major lineages of the genus *Lilium* (Liliaceae) based on nrDNA its sequences, with special emphasis on the Korean Species. J Plant Biol.

[CR15] Li Z, Teng ZH, Li XY, Sui SZ, Li MY (2011). Phylogenetic relationship analysis of 23 Wild species of *Lilium* by SRAP markers. J Agric Biotechnol.

[CR16] Murashige T, Skoog F (1962). A revised medium for rapid growth and bioassays with tobacco tissue culture. Physiol Plant.

[CR17] Nishikawa T, Okazaki K, Uchino T, Arakawa K, Nagamine T (1999). A molecular phylogeny of *Lilium* in the internal transcribed spacer region of nuclear ribosomal DNA. J Mol Evol.

[CR18] Nishikawa T, Okazzki K, Arakawa K, Nagamine T (2001). Phylogenetic analysis of section *Sinomartagon* in genus *Lilium* using sequences of the internal transcribed spacer region in nuclear ribosomal DNA. Breed Sci.

[CR19] Ohkawa K (1989). Time of flower differentiation in lily native to Japan. J Japan Soc Hort Sci.

[CR20] Pelkonen VP, Pirttilä AM (2012). Taxonomy and phylogeny of the genus *Lilium*. Floricult Ornam Biotechnol.

[CR21] Rong L, Lei L, Wang C (2011). Collection and evaluation of the genus *Lilium* resources in northeast China. Genet Resour Crop Evol.

[CR22] Shao JZ, Zhang DC, Yang LZ, Li SM (1994). Study on cytotaxonomy of *Lilium* from Anhui. J Anhui Normal Univ (Nat Sci).

[CR23] Sharma AK, Sharma A (1965). Chromosome technique: theory and practice.

[CR24] Stępiński D (2013). Nucleolar chromatin organization at different activities of soybean root meristematic cell nucleoli. Protoplasma.

[CR25] Stewart RN (1947). The morphology of somatic chromosomes in *Lilium*. Am J Bot.

[CR26] Stover BC, Muller KF (2010). TreeGraph 2: combining and visualizing evidence from different phylogenetic analyses. BMC Bioinform.

[CR27] Sultana S, Lim YP, Bang JW, Choi HW (2011). Internal transcribed spacer (ITS) and genetic variations in *Lilium* native to Korea. Hort Environ Biotechnol.

[CR28] Sun XY (2002). Study on biology of *Lilium callosum*. Master Dissertation, Northeast Forestry University

[CR29] Swofford DL (2002). PAUP*: phylogenetic analysis using parsimony (*and other methods), version 4.0b10 for Macintosh.

[CR30] Thompson JD, Gibson TJ, Plewniak F, Jeanmougin F, Higgins DG (1997). The Clustal X windows interface. Flexible strategies for multiple sequence alignment aided by quality analysis tools. Nucleic Acids Res.

[CR31] van Tuyl JM, Arens P, Ramanna MS, Shahin A, Khan N, Xie SL, Marasek-Ciolakowska A, Lim KB, Barba-Gonzalez R, Kole C (2011). *Lilium*. Wild crop relatives: genomic and breeding resources, plantation and ornamental crops.

[CR32] Yang LP, Ding B, Liu XH, Zhang X (1996). Cytogenetic diversity in *Lilium* L. in northeast China. J Northeast Forest Univ.

[CR33] Yang LP, Zhang XF, Ding B, Zhao LJ (1998). Cultivation of hybrid from *Lilium callosum L*. *regale*. J Northeast Forest Univ.

[CR34] Ying SS, Huang TC (2000). *Lilium* Tourn. ex L. Flora of Taiwan.

[CR35] Zhao F, Xiang DY, Sun XY, Yang LP (2010). Study of flowering biological characteristics of *Lilium callosum*. J Agric Univ Hebei.

[CR36] Zhi LH, Qiu JC, Li XL (2009). Study on the growth progress during the period from germination to blooming of wild lily in Yuxi Mountainous Region. J Zhejiang Agric Sci.

