# Supplementary material for: Re-emergence of Lilium callosum Sieb. et Zucc. in Taiwan after a fire allows propagation and renews the possibility of conservation
Source: Bot Stud. 2017 Nov 13;58:47. doi: 10.1186/s40529-017-0202-x (PMC5684056; doi:10.1186/s40529-017-0202-x)
Supplement: Supplementary file 1 — Additional file 1: Appendix S1. Plants associated with Lilium callosum in the Taiwanese native habitat. [file 40529_2017_202_MOESM1_ESM.docx]

Appendix S1. Plants associated with *Lilium callosum* in the Taiwanese native habitat

| Plant Family | Number | Species |
| --- | --- | --- |
| Anacardiaceae | 1 | *Rhus chinensis* Mill. var. *roxburghiana* (DC.) Rehd. |
| Asclepiadaceae | 1 | *Gymnema sylvestre* (Retz.) Schultes |
| Caprifoliaceae | 1 | *Lonicera japonica* Wall. |
| Compositae | 12 | \| *Ambrosia artemisiifolia* L. \| \| --- \| \| *Artemisia japonica* Thunb. \| \| *Artemisia lancea* Van. \| \| *Aster shimadai* (Kitam.) Nemoto \| \| *Bidens pilosa* L. var. *radiata* Sch. Bip. \| \| *Elephantopus mollis* Kunth \| \| *Erigeron canadensis* L. \| \| *Mikania micrantha* H. B. & K. \| \| *Praxelis clematidea* (Griseb.) R. M. King & H. Robinson \| \| *Stemmacantha uniflora* (L.) M. Dittrich \| \| *Synedrella nodiflora* (L.) Gaertn. \| \| *Syneilesis intermedia* (Hayata) Kitamura \| |
| Cyperaceae | 2 | \| *Fuirena ciliaris* (L.) Roxb. \| \| --- \| \| *Scleria levis* Retz. \| |
| Euphorbiaceae | 4 | \| *Glochidion rubrum* Blume \| \| --- \| \| *Mallotus japonicus* (Thunb.) Muell.-Arg. \| \| *Mallotus repandus* (Willd.) Muell.-Arg. \| \| *Sapium sebiferum* (L.) Roxb. \| |
| Gramineae | 9 | \| *Arundinella hirta* (Thunb.) Tanaka \| \| --- \| \| *Arundinella setosa* Trin. \| \| *Bothriochloa intermedia* (R. Br.) A. Camus \| \| *Bothriochloa ischaemum* (L.) Keng \| \| *Cymbopogon tortilis* (Presl) A. Camus \| \| *Digitaria mollicoma* (Kunth) Henrard \| \| *Eulalia speciosa* (Debeaux) Kuntze \| \| *Paspalum orbiculare* G. Forst. \| \| *Setaria glauca* (L.) P. Beauv. \| |
| Hamamelidaceae | 1 | *Liquidambar formosana* Hance |
| Iridaceae | 1 | *Belamcanda chinensis* (L.) DC. |
| Lauraceae | 1 | *Lindera glauca* (Sieb. & Zucc.) Blume |
| Leguminosae | 1 | *Phyllodium pulchellum* (L.) Desv. |
| Malvaceae | 1 | *Sida cordifolia* L. |
| Passifloraceae | 1 | *Passiflora suberosa* L. |
| Plant Family | Number | Species |
| Rosaceae | 1 | *Rosa bracteata* Wendl. |
| Rubiaceae | 2 | \| *Morinda parvifolia* Bartl. \| \| --- \| \| *Mussaenda parviflora* Matsum. \| |
| Rutaceae | 1 | *Zanthoxylum nitidum* (Roxb.) DC. |
| Sterculiaceae | 1 | *Helicteres angustifolia* L. |
| Styracaceae | 1 | *Styrax matsumuraei* Perkins |
| Symplocaceae | 1 | *Symplocos chinensis* (Lour.) Druce |
| Tiliaceae | 1 | *Grewia rhombifolia* Kanehira & Sasaki |
| Ulmaceae | 1 | *Celtis sinensis* Pers. |
| Umbelliferae | 1 | *Bupleurum kaoi* Liu & Chao & Chuang |
| Verbenaceae | 1 | *Vitex negundo* L. |
| Zingiberaceae | 1 | *Alpinia zerumbet* (Pers.) B. L. Burtt & R. M. Smith |
